# HER3 Expression Is a Marker of Tumor Progression in Premalignant Lesions of the Gastroesophageal Junction

**DOI:** 10.1371/journal.pone.0161781

**Published:** 2016-08-25

**Authors:** Brett L. Ecker, Laura Taylor, Paul J. Zhang, Emma E. Furth, Gregory G. Ginsberg, Matthew T. McMillan, Jashodeep Datta, Brian J. Czerniecki, Robert E. Roses

**Affiliations:** 1 Department of Surgery, University of Pennsylvania, Philadelphia, PA, United States of America; 2 Department of Pathology and Laboratory Medicine, University of Pennsylvania, Philadelphia, PA, United States of America; 3 Department of Gastroenterology, University of Pennsylvania, Philadelphia, PA, United States of America; Shiga University of Medical science, JAPAN

## Abstract

Overexpression of receptor tyrosine kinases (RTK), including members of the HER family, has prognostic and therapeutic significance in invasive esophagogastric carcinoma. RTK expression in premalignant gastroesophageal lesions has not been extensively explored. Formalin-fixed paraffin-embedded tissue samples of esophageal biopsy specimens from 73 patients with Barrett’s esophagus with either low-grade dysplasia (LGD) (n = 32) or high-grade dysplasia (HGD) (n = 59) were analyzed for HER1, HER2, HER3 and CMET expression by immunohistochemistry (IHC). Immunophenotype was correlated with histologic and clinical features. High-grade dysplasia (HGD) was associated with overexpression of HER1 (20.7% vs. 3.1%, p = 0.023), HER2 (5.3% vs. 0.0%, p = 0.187) and HER3 (47.4% vs. 9.4%, p<0.001) compared to low-grade dysplasia (LGD). There was a significant association of HER2 (20.0% vs. 2.1%, p = 0.022) and HER3 (80.0% vs. 40.4%, p = 0.023) overexpression in HGD lesions associated with foci of invasive carcinoma compared to those without invasive foci. Overexpression of CMET was observed in 42.9% of specimens, was increasingly observed with HGD compared to LGD (58.3% vs. 36.7%, p = 0.200), and was most often co-expressed with HER3 (62.5% of HER3-positive specimens vs. 38.2% of HER3-negative specimens, p = 0.212). In summary, HER3 is frequently overexpressed in high-grade dysplastic lesions of the gastroesophageal junction and may be a marker of invasive progression. These data provide rationale for targeting HER2 and HER3 pathways in an early disease setting to prevent disease progression.

## Introduction

Barrett's esophagus (BE), or the presence of metaplastic columnar epithelium in the distal esophagus, predisposes to the development of esophageal adenocarcinoma (EAC) [1]. While the histologic transition from dysplasia to invasive malignancy is well characterized, carcinogenesis in metaplastic cells involves genetic alterations that are incompletely understood. There is a clinical need for biomarkers to predict the likelihood of progression from dysplasia to invasive carcinoma. Next-generation sequencing studies of both EAC and BE have identified several candidate genes for further exploration [2]. Additionally, recent reports have identified receptor tyrosine kinase (RTK) expression, specifically human epidermal growth factor receptor (HER)-2, in a subset of dysplastic Barrett’s lesions where the rate of HER2 expression correlated with degree of dysplasia, implicating related pathways in tumorigenesis [3–5].

Homo- and hetero-dimerization of HER receptors drive signal activation, and clustered overexpression of multiple members of the HER family have been observed in esophagogastric carcinoma [6–8]. Overexpression of the hepatocyte growth factor receptor (CMET) has also been correlated with poor prognosis in esophageal adenocarcinoma, and inhibition of CMET-dependent signaling regulates the activity of HER1 and HER3 [9]. Whereas HER2 overexpression has been targeted with trastuzumab for gastric and gastroesophageal junction carcinomas in the metastatic setting with a modest impact on outcome [10], little is known about the clustered overexpression of RTKs and the potential of RTK-targeted therapies for dysplastic Barrett’s lesions.

Given the significant cross interactions among the HER family members and CMET, we performed an exploratory analysis of expression of these proteins in dysplastic lesions of the gastroesophageal junction. We aimed to characterize RTK expression in efforts to characterize the molecular signatures of dysplasia and identify opportunities for novel treatment approaches.

## Patients and Methods

Following approval by the University of Pennsylvania institutional review board (protocol #819273), the clinical records and histologic specimens from 73 patients with Barrett’s esophagus with dysplasia between 2003 and 2012 were reviewed retrospectively. Consent was not required, as patient information was anonymized and deidentified prior to analysis. Patients who underwent serial endoscopies with repeat biopsies as part of a continued surveillance program at our institution and had available pathology were included in this analysis. If multiple biopsies were taken at the time of any given endoscopy, a representative slide was chosen for immunohistochemistry.

**Fig 1:**
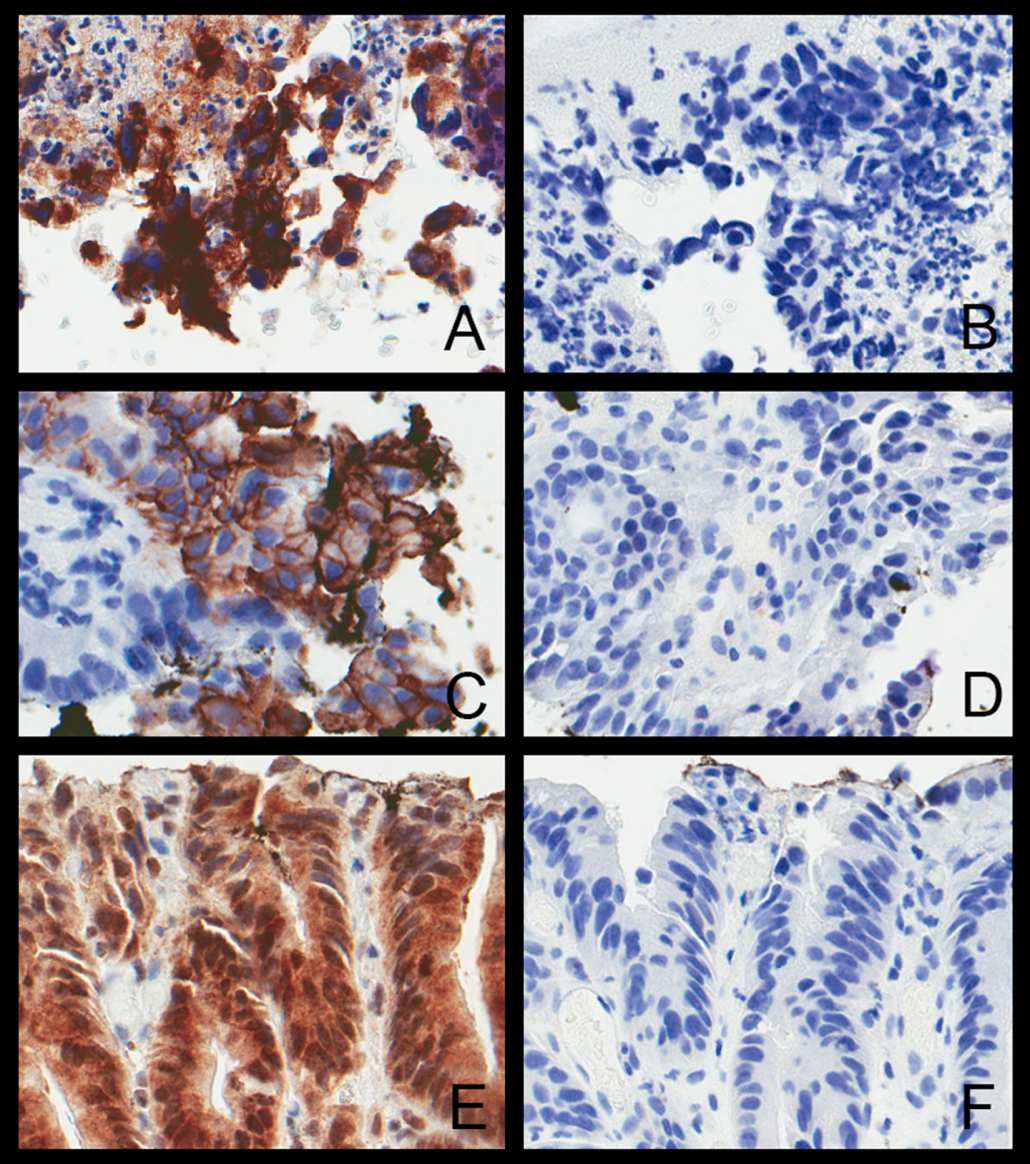
Immunohistochemisty scoring of HER staining. Representative high grade dysplasic Barretts esophagus with (A) 3+ HER1, (C) 3+ HER2, and (E) 3+ HER3. Positive and negative controls were used in all instances (negative controls for HER1, HER2 and HER3 are depicted in B, D, and F, respectively).

Formalin-fixed paraffin-embedded tissue blocks from stored endoscopic biopsy and mucosal resection specimens were sectioned at 5μm on plus slides (Fisher Scientific) and subsequently deparaffinized and rehydrated. The diagnosis of low-grade dysplasia was confirmed by two pathologists. Immunohistochemical staining for HER2 (HercepTest, DAKO) was performed on the DAKO Autostainer (DAKO, Carpinteria, CA); EGFR (clone H11; 1:50; DAKO) and HER3 (clone RTJ.2; 1:30; Santa Cruz Biotechnology) were performed on the Leica Bond-III instrument. HER oncoprotein expression was evaluated following the score system suggested by FDA guidelines [11]; the immunoreaction was scored as follows: 3+ = complete and intense membrane staining of >10% cells; 2+ = complete but moderate staining of >10% cells, 1+ = weak and incomplete staining in>10% cells; 0 = no membrane staining, or staining in <10% cells. Membrane 3+ HER staining was considered positive, as was membrane 2+ HER2 staining in >10% of tumor cells [10,12] (**Fig 1**). IHC antibodies were previously validated [12,13]. and positive staining controls were checked with each use. CMET immunohistochemistry was performed in 42 cases when sufficient tissue was available; moderate or strong membranous staining in ≥50% of tumor cells was considered positive [14]. RTK overexpression was correlated with clinical data to evaluate for associations with invasive carcinoma, either paired dysplasia-adenocarcinoma biopsy specimens or the diagnosis of adenocarcinoma on subsequent biopsy specimens. Carcinoma was distinguished from HGD by the presence of invasion, at least into the lamina propria, in accordance with AJCC 7^th^ edition TNM staging [15]. The clinical variables analyzed included age, gender, race (Caucasian, African American or Black, Asian, or other), alcohol (≥14 standard drinks/week amongst men and ≥7 standard drinks/week amongst women [16]) and smoking intake, and a family history of solid organ malignancy.

### Statistical Analysis

Descriptive statistics are presented as frequencies for categorical variables and median (interquartile range [IQR]) for continuous variables. Pearson’s χ^2^ or Fisher’s exact tests and Wilcoxon rank-sum test were used to analyze categorical and continuous variables, respectively. P-values ≤0.05 were considered statistically significant; all tests were two-sided. Analyses were carried out using SPSS v22.0 (IBM Corp., Armonk, NY).

## Results

### Patient Demographics

A total of 73 patients with Barrett’s esophagus with low-grade dysplasia (n = 32) or high-grade dysplasia (n = 59) were identified. IHC was performed on a single biopsy specimen for most (80.4%) of the study cohort; the remaining patients (19.6%) underwent serial endoscopies as part of a continued surveillance program and had time-distinct pathology specimens available to include in this analysis. Median age of the cohort was 65 years (IQR 60–73 years); 81.9% were male and 87.5% were Caucasian. The rate of alcohol use in the cohort was 14.3% and the rate active cigarette use was 6.3%, yet 55.6% were former smokers. Over one-quarter (26.4%) had a family history of solid organ malignancy. Thirty-two patients (47.1%) had endoscopic evidence of a hiatal hernia and 3 patients (4.3%) had previously undergone surgical fundoplication. Medical acid suppression prior to the index endoscopy was documented in 42 patients (80.8%). The majority of patients (n = 37; 56.1%) had short segment BE while 29 patients (43.9%) had long (≥5 cm) segment BE; only 15 patients had a documented Prague classification. There were no significant differences between the LGD and HGD cohorts in the measured clinical and demographic variables (**Table 1**). There was an increased rate of long segment Barretts esophagus (50.0% vs. 31.8%, p = 0.161) and a history of prior endoscopic therapies (31.1% vs. 9.1%, p = 0.047) in the HGD cohort.

**Table 1:** Demographic and Clinical Characteristics of Cohort with Dysplastic Barrett’s Esophagus, and Univariate Comparison of Low-grade and High-grade Dysplastic Patients. (n = 73)Abbreviations: LGD, low-grade dysplasia; HGD, high-grade dysplasia.

Median (IQR) or no. of patients (%)		LGD	HGD	p-value
Age, years		64.0 (60.0–77.5)	66.0 (63.0–72.0)	0.621
Sex, male		19 (73.1)	41 (87.2)	0.130
Caucasian race		23 (95.8)	41 (93.2)	0.755
Cigarette use	Current	2 (9.1)	2 (4.8)	0.681
	Former	13 (59.1)	23 (54.8)	
Alcohol use		2 (9.1)	7 (16.7)	0.683
Positive family history		6 (28.6)	13 (32.5)	0.753
Hiatal hernia		10 (45.5)	22 (47.8)	0.855
History of fundoplication		1 (4.3)	2 (4.3)	1.000
Proton pump inhibitor use		14 (73.7)	28 (84.8)	0.325
Barrett’s length	Short	15 (68.2)	22 (50.0)	0.161
	Long	7 (31.8)	22 (50.0)	
Previous therapy		3 (13.0)	14 (31.1)	0.104
Endoscopic resection/ablation		2 (8.7)	14 (31.1)	--
Surgical resection		1 (4.3)	0 (0.0)	

### Comparative Immunohistochemical Analysis of Barrett’s Esophagus with Low-Grade vs. High-Grade Dysplasia

**Fig 2:**
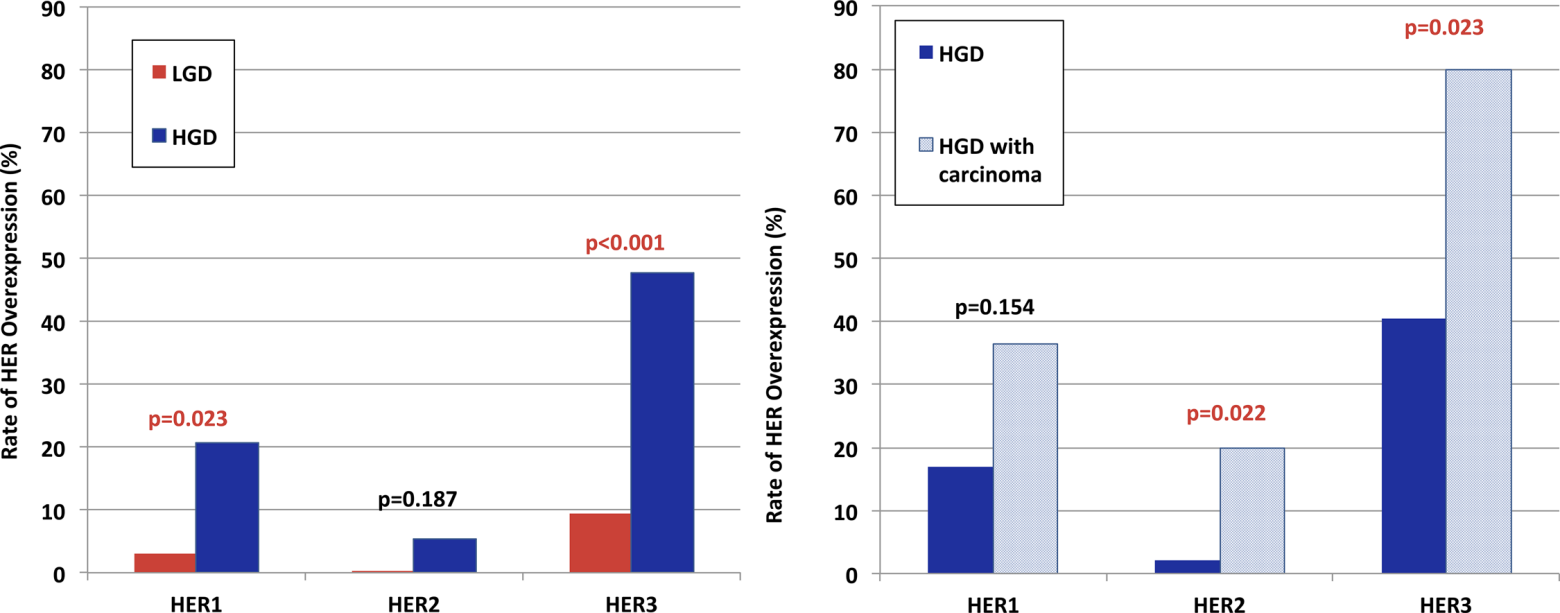
Rate of HER family overexpression in (A) Barrett’s esophagus with low- or high-grade dysplasia, and (B) high-grade dysplastic Barrett’s lesions with or without associated invasive cancer.

The rate of HER overexpression was 14.4%, 3.4% and 33.7% for HER1, HER2 and HER3, respectively. HGD was associated with a significantly increased rate of HER1 overexpression (20.7% vs. 3.1%, p = 0.023) and HER3 overexpression (47.4% vs. 9.4%, p<0.001) compared to LGD. There was a nonsignificant trend toward increased overexpression of HER2 in HGD lesions (5.3% vs. 0.0%, p = 0.187) (**Fig 2**).

By exploratory analysis, an additional 3 patients with esophageal adenocarcinoma at initial biopsy were identified to evaluate the rate of HER family overexpression. The rate of HER overexpression was 33.3%, 0.0% and 66.7% for HER1, HER2 and HER3, respectively.

### Risk of Invasive Carcinoma in the Setting of Dysplasia

Foci of invasive esophageal adenocarcinoma were associated with dysplastic lesions in 6 cases (HGD: 10.2% vs. LGD: 0.0%, p<0.001); an additional 5 patients were diagnosed with invasive adenocarcinoma on subsequent biopsy specimens (HGD: 8.5% vs. LGD: 0.0%, p = 0.090). There was an absolute increase in HER1 (36.4% vs. 17.0%, p = 0.154) overexpression in HGD lesions associated with carcinoma compared to those without evidence of invasion. There was a significant increase in HER2 expression (20.0% vs. 2.1%, p = 0.022) and HER3 expression (80.0% vs. 40.4%, p = 0.023) in HGD lesions associated with carcinoma. Patients who developed esophageal adenocarcinoma most commonly had AJCC stage I disease (n = 10; 90.9%); one patient (9.1%) developed AJCC stage II disease. The management of these patients included: esophagectomy (n = 4; 40.0%) or endoscopic therapies (n = 6; 60.0%).

Patients were followed for a median of 47 (IQR 22–65) months. An additional 4 patients, all previously with HGD, developed invasive carcinoma. The median time to diagnosis of EAC was 23 (IQR 16.5–35.5) months; a median of 6.5 (IQR 6.0–7.75) subsequent endoscopies was performed prior to diagnosis of EAC. There was an absolute increase in HER1 (36.4% vs. 0.0%, p = 0.159), HER2 (20.0% vs. 0.0%, p = 0.334) and HER3 (80.0% vs. 50.0%, p = 0.262) overexpression in HGD lesions associated with early EAC as compared to late occurrence.

### CMET Expression in Dysplastic Barrett’s Esophagus

Overexpression of CMET was identified in 18 of 42 (42.9%) evaluated specimens and increasingly observed in HGD compared to LGD specimens (58.3% vs. 36.7%, p = 0.200). CMET was co-expressed in 62.5% of HER3-positive specimens compared to 38.2% of HER3-negative specimens (p = 0.212). Similar trends were not observed in HER1-positive (p = 0.729) or HER2-positive (p = 1.0) specimens.

## Discussion

This analysis of RTK expression in dysplastic lesions of the gastroesophageal junction confirms that (1) HER family proteins are upregulated in Barrett’s esophagus with dysplasia; (2) the frequency of HER family overexpression is positively correlated with the degree of dysplasia; and (3) HER protein upregulation, particularly HER2 and HER3, in dysplastic lesions is associated with an increased incidence of associated invasive cancer.

Previous studies of RTK expression in Barrett’s esophagus have been limited to an assessment of HER2, which is overexpressed in a minority of cases [3–5]. HER2 overexpression in this study was present in 3.4% of biopsy specimens, lower than the rate of HER1 or HER3 overexpression. This pattern is consistent with HER family protein expression in invasive gastroesophageal junction cancers, where HER3 is overexpressed more commonly than HER2 [7]. In *pre-invasive* lesions, increasing HER3 protein overexpression with progression from LGD to HGD and frequent overexpression of HER3 in particular, represent novel, though not unanticipated findings. Moreover, the increase in clustered overexpression of HER receptor tyrosine kinases from LGD to HGD to the small sample of invasive EAC suggests a role in tumorigenesis.

In addition to serving as a dimerization partner of HER1 and HER2, HER3 has been implicated in tumor cells’ acquisition of resistance to targeted therapy. HER3-mediated activation of phosphoinositide 3-kinase (PI3K)/Akt signaling correlates with tumor sensitivity to HER-family RTK inhibitor therapy. CMET activates HER3 tyrosine phosphorylation and PI3K activation independent of HER1 and HER2, which has been associated with gefitinib resistance in preclinical models [17]. Unfortunately, the nonsignificant co-expression of HER3 and CMET in this study cannot provide further support for the possible interplay between these receptors.

The present data have several clinical implications. HER2 and HER3 may serve as biomarkers for occult invasive disease in patients with Barrett’s esophagus and HGD. Endoscopic resection and/or ablative techniques have been introduced to mitigate the morbidity of esophagectomy, particularly in the management of high-grade dysplasia [18,19]. Increased endoscopic surveillance or earlier referral for surgical evaluation may be warranted in this subset of tumors with more aggressive biology.

The present data also suggest an opportunity for targeted secondary prevention. Targeted immune conditioning against HER2 in early breast cancer has been shown to result in consistent immune sensitization and frequent clinical responses [20,21]. Such an approach remains a more distant goal for gastrointestinal malignancies. Notwithstanding, current endoscopic or surgical treatment options for Barrett’s esophagus all have significant limitations. Alternative strategies that spare morbidity and mitigate the risk of invasive carcinoma are needed. In subsets of patients with Barrett’s esophagus with dysplasia, therapeutics targeting HER2 or HER3 may be explored as a means of forestalling or preventing gastroesophageal carcinoma.

There are significant limitations of the present data. While dysplasia is an effective biomarker of malignant potential in Barrett’s esophagus [1], diagnostic accuracy is limited by biopsy sampling error and interobserver disagreement, which may be occur in up to 15% of cases for high-grade dysplasia [22]. This may explain, in part, the lower rate of HER2 expression in this study, as compared to a previously published analysis [3,4]. Second, the results cannot be extrapolated to the setting of Barrett’ esophagus without dyplasia. The clinical goal of this research was to identify a high-risk cohort of patients with dysplasia for future experimental therapeutics. Barrett’s dysplasia was specifically targeted because of the increased cancer risk yet unpredictable pattern of disease progression. Assuming that cancers develop via the progression of Barrett’s esophagus to dysplasia to invasive carcinoma, we focused on dysplastic lesions. This allowed for more frequent occurrence of our clinical outcome (i.e. invasive progression) than would have been observed with all cases of Barrett’s esophagus without dysplasia. Proper identification of high-risk patients and appropriate molecular targets provides the theoretical opportunity to intervene in a clinically meaningful way.

Additionally, the possibility of type 1 and/or type 2 errors cannot be excluded, given the size of the patient sample. Since it is the first of its kind to report upon HER1 or HER3 overexpression in dysplastic Barrett’s esophagus, there is no standard clinically significant difference in the proportion of patient with overexpression of these RTKs upon which to base a power calculation. Nevertheless, this study is a hypothesis-generating exploratory analysis that provides clinically relevant biomarkers for external validation.

## Conclusions

The present data indicate that multiple HER family proteins are upregulated in dysplastic lesions of the gastroesophageal junction and suggests a relationship between HER3 expression in dysplastic lesions and malignant transformation. These findings may justify a more aggressive management approach for HER-2 and HER3-expressing dysplastic lesions and provide rationale for the future application of RTK-targeted therapeutics in an early disease setting.
